# Postoperative Morbidity Following Loop Ileostomy Reversal after Primary Elective or Urgent Surgery: A Retrospective Study with 145 Patients

**DOI:** 10.3390/jcm12020452

**Published:** 2023-01-05

**Authors:** Roberto Peltrini, Giuseppe Magno, Daniela Pacella, Biancamaria Iacone, Antonia Rizzuto, Umberto Bracale, Francesco Corcione

**Affiliations:** 1 Department of Public Health, Federico II University Hospital, 80131 Naples, Italy; 2 Department of Medical and Surgical Science, University Magna Græcia of Catanzaro, 88100 Catanzaro, Italy; 3 Department of Advanced Biomedical Sciences, Federico II University Hospital, 80131 Naples, Italy

**Keywords:** ileostomy reversal, loop ileostomy, postoperative complications, colorectal surgery

## Abstract

Temporary loop ileostomy is usually performed to protect distal anastomosis or to treat urgent surgical cases. The aim of this study is to evaluate whether, after primary urgent stoma construction, patients undergoing ileostomy reversal have different postoperative outcomes compared with patients who have protective stoma performed in an elective setting. A retrospective observational study was conducted including patients who underwent ileostomy reversal. Baseline patient characteristics and perioperative outcomes were collected in a single database. The overall morbidity rate during recovery was fixed as a primary outcome. Between 2011 and 2021, the complete data records of 145 patients were evaluated. After ileostomy reversal, the postoperative morbidity rate did not differ between groups (14.4% vs. 11.5%, *p* = 0.790). Even considering each complication, such as ileus, small bowel obstruction, bleeding and wound infection, no significant difference was detected. Similarly, the time to first flatus was 2.25 ± 1.24 vs. 2.1 ± 0.99 (*p* = 0.379) and the length of hospital stay was 5.43 ± 3.03 vs. 5.84 ± 5.15 (*p* = 0.568). The only significant factor associated with postoperative complications on logistic regression analysis was the presence of comorbidities (OR 4.49; 95% CI 1.19–29.4, *p* = 0.05). In the present cohort of patients, there was no difference in the postoperative complication rate after stoma closure following elective or urgent indication for surgery.

## 1. Introduction

Anastomotic leakage (AL) is the most feared complication after Total Mesorectal Excision (TME) for rectal cancer, occurring up to 20% of cases [1]. It is associated not only with considerable postoperative morbidity, mortality [2] and impaired functional outcomes [3], but also with poor long-term oncological outcomes [4,5]. Protective loop ileostomy is often used in an elective setting for high-risk anastomosis to reduce the incidence and severity of clinically significant AL [6,7]. Additionally, proximal diversion can be used for the treatment of emergency postoperative complications when anastomotic dehiscence, bowel occlusion or perforation occur. In this context, ileostomy is often considered temporary and patients usually undergo stoma reversal 8–12 weeks after previous intervention. However, ileostomy closure and the restoration of bowel continuity are not without complications [8,9]. Postoperative morbidity after ileostomy reversal may be affected by the primary surgical approach [10,11], the interval between the previous operation and ileostomy closure [12,13], the anastomotic technique [14] and the use of laparoscopy [15,16]. Furthermore, even different indications for ileostomy construction, in the elective or urgent setting, may influence recovery. However, the literature data are limited in this regard. Therefore, the aim of this study is to evaluate if patients undergoing ileostomy reversal after primary urgent stoma construction have different postoperative outcomes compared with patients who have protective stoma performed in an elective setting.

## 2. Materials and Methods

A retrospective review of patients who underwent ileostomy reversal between 2011 and 2018 at Monaldi Hospital and between 2018 and 2021 at Federico II University Hospital in Naples, Italy, was performed. Diverting loop ileostomy was performed both during elective colorectal surgery or during urgent surgery (primary emergencies or surgical treatment of postoperative complications). As we assume a more complex ileostomy reversal after bowel perforation and occlusion due to adhesions and a potential high rate of open surgery, we fixed overall morbidity rate during recovery as primary outcome. Demographic and clinical information such as age, gender, BMI, ASA score, previous surgery and comorbidities, time to stoma closure and postoperative outcomes were collected in a single database. Incomplete medical records were excluded from the study. Ileus was defined as delayed return of bowel function, with abdominal distension, nausea or intolerance of oral diet [17]. Small bowel obstruction was defined as the combination of both clinical symptoms and radiographic evidence of dilated bowel with clear obstruction [18]. Furthermore, only postoperative bleeding that required transfusions was considered.

### 2.1. Surgical Technique

All patients who underwent ileostomy closure received general anesthesia and prophylactic intravenous antibiotics. A circumferential peristomal incision was performed and deepened to the fascia. The intestinal segments were adequately mobilized reaching the abdominal cavity. The stoma was resected and an isoperistaltic or antiperistaltic side-to-side stapled anastomosis was performed. The abdominal wall was closed in two layers using continuous absorbable sutures and the skin incision was closed with interrupted stitches or partially left open using a purse-string suture depending on the surgeon’s preference. Before closure, patients who had received previous surgery with a minimally invasive approach had laparoscopy to take down intra-abdominal adhesions as needed [17].

### 2.2. Statistical Analysis

Data are presented as frequency (percentages) for the categorical variables, while they are presented as mean ± standard deviation for continuous variables. Comparisons between the two groups are performed using Student’s *t*-test or with Mann–Whitney U test, as appropriate. Comparisons between groups for categorical variables are performed with the chi-square test or with Fisher’s exact test as appropriate. For all analyses, the significance level was set at α = 0.05. All analyses were performed using the statistical software R, version 4.0.3. Associations between possible predictive factors and postoperative complications were explored using logistic regression models and results were reported as OR with 95% confidence intervals. Odds ratios (ORs) were obtained exponentiating the regression coefficients.

## 3. Results

A total of 145 patients who underwent ileostomy reversal were identified. Seventy-six patients who had a protective ileostomy during elective colorectal surgery were compared with sixty-nine patients who had ileostomy as a surgical treatment in an urgent setting, including anastomotic leakage (*n* = 48), bowel occlusion (*n* = 7), postoperative bowel perforation (*n* = 9), iatrogenic colonoscopy perforation (*n* = 3), bowel perforation following a urologic procedure (*n* = 1) and complicated abdominal tuberculosis (*n* = 1). The patient demographics and characteristics of primary surgery are shown in Table 1. A significant difference was found in terms of primary diagnosis (*p* = 0.005), associate comorbidities such as hypertension (*p* = 0.041) and diabetes (*p* = 0.007) and previous surgery (*p* < 0.001). The time to stoma closure was significantly longer in the urgent group (141 ± 115 vs. 190 ± 168 days, *p* = 0.049). Furthermore, previous surgery with a laparoscopic approach was more frequent during elective surgery whereas the open approach was more frequent during urgent surgery (*p* = 0.035). After ileostomy reversal, the postoperative morbidity rate did not differ between groups (14.4% vs. 11.5%, *p* = 0.790) (Table 2). Even considering each complication, such as ileus, small bowel obstruction, bleeding and wound infection, no significant difference was detected. Similarly, the time to first flatus was 2.25 ± 1.24 vs. 2.1 ± 0.99 (*p* = 0.379) and the length of hospital stay was 5.43 ± 3.03 vs. 5.84 ± 5.15 (*p* = 0.568). The only significant factor associated with postoperative complications on logistic regression analysis (Table 3) was the presence of comorbidities (OR 4.49; 95% CI 1.19–29.4, *p* = 0.05).

## 4. Discussion

Although protective loop ileostomy after TME could be avoided in selected patients using specific intraoperative strategies [19,20], it still has an important role in reducing the incidence and severity of the anastomotic leakage [6,7]. The short-term outcomes after diverting loop ileostomy closure can be affected by several factors. However, the impact of primary colorectal surgery as a predictor of postoperative complications after ileostomy reversal was addressed only in a recent article [21]. To the best of our knowledge, a direct comparison between elective or urgent settings at first surgery is missing in the literature. The present study shows that the postoperative complication rate following ileostomy reversal does not differ between previous urgent or elective surgery.

The overall morbidity rate after ileostomy closure is approximately 20% [9,22], and small bowel obstruction is the most common complication [23,24]. Intra-abdominal adhesions arise from peritoneal trauma involving several cellular, biochemical and immunological factors. They develop when abnormal scar tissue occurs between two contiguous peritoneal surfaces. As laparoscopy reduces surgical trauma, abdominal wall incisions and tissue manipulation, it is associated with decreased small bowel obstructions due to adhesions compared with open surgery [25]. However, not only the surgical approach but also pathologic conditions can have a significant influence on the development of adhesions such as bowel perforation with peritonitis. The inflammation of the peritoneal surface can lead to fibrotic tissue with localized or generalized immunobiological tissue reactions.

The benefits of a minimally invasive approach before stoma closure in terms of ileus and overall complication are well documented. In fact, recent data from the Cleveland Clinic show that ileostomy reversal after laparoscopic colorectal surgery is associated with a shorter hospital stay, postoperative ileus and overall complication rate than open surgery [10]. Although we found that a minimally invasive approach was more common in the elective surgery group, this significant difference had no impact on the postoperative morbidity rate. Specifically, ileus and small bowel obstruction did not differ between groups. In the present study, 66.6% of previous urgent surgery was performed by laparoscopy. This may have reduced the development of postoperative adhesions, which remain limited to post-inflammatory fibrosis only.

The timing of temporary ileostomy reversal can influence recovery, with worse outcomes when performed after 90 days [12]. In this regard, early closure (within 30 days) may have some advantages compared with delayed closure in selected patients [26]. We found a longer time to stoma closure in the urgent setting group did not affect outcomes.

This study has several limitations, such as its retrospective design. The results should be interpreted with caution, due to both the selection bias and the limited sample size. We found a significant difference in some of the patients’ preoperative characteristics. In order to reduce the heterogeneity between cohorts and increase sample size, multicenter studies with matched groups are warranted.

## 5. Conclusions

In the present cohort of patients, a difference in postoperative complication rate after stoma closure following elective or urgent surgery was not detected. These results help to provide more accurate information during preoperative patient counselling and avoid an undue delay in restoring digestive continuity after urgent surgery. Further studies are warranted to support these findings.

## Figures and Tables

**Table 1:** Baseline characteristics of the study population.

	Total (*n* = 145)	Elective Surgery (*n* = 76)	Urgent Surgery (*n* = 69)	*p* Value
Age, years (mean ± SD)	64.49 ± 13	65.32 ± 14	63.5 ± 13	0.370
Gender, *n* (%) Male Female	82 (56.5) 63 (43.4)	40 (52.6) 36 (47.3)	42 (60.8) 27 (39.1)	0.406
BMI (mean ± SD)	24.4 ± 4.5	24.55 ± 4.4	24.2 ± 4.6	0.643
ASA, *n* (%) I—II III—IV	109 (75) 36 (25)	57 (75) 19 (25)	52 (75.3) 17 (24.6)	>0.999
Diagnosis, *n* (%) Benign Malignant	42 (28.9) 103 (71.0)	12 (15.7) 64 (84.2)	26 (37.6) 43 (62.3)	0.005
Comorbidities, *n* (%) Hypertension Cardiovascular disease Chronic obstructive pulmonary disease Diabetes Chronic kidney disease Overall patients with comorbidities, *n* (%)	60 (41.3) 34 (23.4) 7 (4.8) 16 (11.0) 9 (6.2) 96 (66.2)	38 (50) 21 (27.6) 2 (2.6) 14 (18.4) 5 (6.5) 54 (71.0)	22 (31.8) 13 (18.8) 5 (7.2) 2 (2.8) 4 (5.7) 42 (60.8)	0.041 0.293 0.348 0.007 >0.999 0.263
Time to stoma closure, days (mean ± SD)	164 ± 144	141 ± 115	190 ± 168	0.049
Previous surgery: Open *n* (%) Lap *n* (%)	32 (77.9) 113 (22.0)	11 (14.4) 67 (88.1)	21 (30.4) 46 (66.6)	0.035
Type of anastomosis *n* (%) isoperistaltic antiperistaltic	99 (68.2) 46 (31.7)	51 (67.1%) 25 (32.8)	48 (69.5) 21 (30.4)	0.889
Associated procedures *n* (%) Incisional hernia repair others	25 (17.2) 2 (1.3)	10 (13.1) 1 (1.3)	15 (21.7) 1 (1.4)	0.252 0.907

Standard Deviation (SD); American Society of Anesthesiologists (ASA); Body Mass Index (BMI).

**Table 2:** Postoperative outcomes.

	Total (*n* = 145)	Elective Surgery (*n* = 76)	Urgent Surgery (*n* = 69)	*p* Value
Time to first flatus. days (mean ± SD)	2.17 ± 1.13	2.25 ± 1.24	2.1 ± 0.99	0.379
Overall morbidity, *n* (%)	19 (13.1)	11 (14.4)	8 (11.5)	0.790
Ileus	8 (5.5)	4 (5.2)	4 (5.8)	>0.999
Small bowel obstruction	3 (2)	2 (2.6)	1 (1.4)	>0.999
Bleeding	4 (2.7)	3 (3.9)	1 (1.4)	0.622
Wound infection	1 (2.2)	1 (1.3)	0	>0.999
Others	3 (2)	1 (1.3)	2 (2.9)	0.605
Overall patients with comorbidities, *n* (%)	17 (11.72)	9 (11.8)	8 (11.5)	>0.999
LOS, days (mean ± SD)	5.63 ± 4.16	5.43 ± 3.03	5.84 ± 5.15	0.568

Length of stay (LOS). Standard Deviation (SD).

**Table 3:** Predictive factors of postoperative complications identified by logistic regression analysis.

	OR	95% CI	*p*-Value
time_to_stoma_closure:			
<90	—	—	
≥90	1.95	0.65, 7.23	0.266
Previous_surgery:			
lap	—	—	
open	2.14	0.68, 6.19	0.169
Diagnosis:			
benign	—	—	
malignant	0.54	0.19, 1.58	0.243
Comorbidities:			
no	—	—	
yes	4.49	1.19, 29.4	0.05
Indication for surgery:			
elective	—	—	
urgent	0.98	0.35, 2.71	0.963

## Data Availability

The data presented in this study are available on request from the corresponding author.
